# Prognostic determinants of hearing outcomes in children with congenital cytomegalovirus infection

**DOI:** 10.1038/s41598-022-08392-w

**Published:** 2022-03-25

**Authors:** Ta-Hsuan Lo, Pei-Hsuan Lin, Wei-Chung Hsu, Po-Nien Tsao, Tien-Chen Liu, Tzong-Hann Yang, Chuan-Jen Hsu, Li-Min Huang, Chun-Yi Lu, Chen-Chi Wu

**Affiliations:** 1 Department of Otolaryngology, National Taiwan University Hospital, Address: 7, Chung-Shan S. Rd., 10002 Taipei, Taiwan, ROC; 2 Department of Otolaryngology, National Taiwan University Biomedical Park Hospital, Hsinchu, Taiwan; 3 Department of Otolaryngology, National Taiwan University Hospital Yunlin Branch, Yunlin, Taiwan; 4 Department of Pediatrics, National Taiwan University Hospital, Address: 7, Chung-Shan S. Rd., 10002 Taipei, Taiwan, ROC; 5 Department of Otorhinolaryngology, Taipei City Hospital, Taipei, Taiwan; 6 Department of Otolaryngology, Taichung Tzu-Chi Hospital, Taichung, Taiwan; 7 Department of Medical Genetics, National Taiwan University Hospital, Taipei, Taiwan; 8 Graduate Institute of Clinical Medicine, National Taiwan University College of Medicine, Taipei, Taiwan; 9 Department of Medical Research, National Taiwan University Biomedical Park Hospital, Hsinchu, Taiwan

**Keywords:** Auditory system, Risk factors

## Abstract

Congenital cytomegalovirus (cCMV) infection is the most prevalent cause of non-genetic sensorineural hearing loss (SNHL) in children. However, the prognostic determinants of SNHL remain unclear. Children with cCMV infection in a tertiary hospital were enrolled. The presence of cCMV-related symptoms at birth, the newborn hearing screening (NHS) results, and the blood viral loads were ascertained. Audiologic outcomes and initial blood viral loads were compared between different groups. Of the 39 children enrolled, 16 developed SNHL. SNHL developed in 60% of children who were initially symptomatic, and in 34.5% of those who were initially asymptomatic with normal hearing or isolated hearing loss, respectively. Failuire in NHS was a reliable tool for early detection of SNHL. The initial viral loads were higher in children who were symptomatic at birth, those who failed NHS, and those who developed SNHL. We observed SNHL deterioration in a patient after CMV DNAemia clearance was achieved, and in another patient with the flare-up of viral load. The presence of cCMV-related symptoms at birth, failure in NHS, and blood viral load might be the prognostic factors for hearing outcomes. Regular audiologic examinations are necessary in all children with cCMV infection even after CMV DNAemia clearance.

## Introduction

Sensorineural hearing loss (SNHL) is the most common sensory deficit in children. It is estimated that the prevalence of SNHL at birth was 0.186% in United States^1^. Pediatric SNHL is caused by a plethora of genetic and acquired etiologies, and at least 50–60% of childhood SNHL in developed countries are attributed to genetic etiologies^1–4^. Among the acquired etiologies, congenital cytomegalovirus (cCMV) infection is most prevalent, accounting for 10–20% of childhood hearing loss^1,3^.

In infants with cCMV infection, approximately 10–15% are symptomatic at birth, whereas the remaining majority of infected newborns are asymptomatic at birth^3,5–10^. SNHL can occur in both symptomatic and asymptomatic newborns. Of the newborns with symptomatic and asymptomatic cCMV infection, 22%–65% and 6%-23%, respectively, eventually develop SNHL^7,9–13^. SNHL caused by cCMV infection can be unilateral or bilateral, fluctuating, progressive, or delayed-onset ^10,12–14^.

SNHL is the most common long-term sequela in children with cCMV infection^5,9,15^. The presentation, severity, and progression of SNHL resulting from cCMV infection are highly variable^7,10,12,16^. Unfortunately, it is still difficult to predict which of the cCMV-infected infants will develop SNHL and how severe their SNHL will be^10,17^. Some studies reported that an increased cytomegalovirus (CMV) viral burden in peripheral blood during infancy might be associated with increased risk for SNHL^18–20^. However, the association between CMV viral load and SNHL was not consistently seen in other studies^10,19,21–24^. Moreover, there is still a paucity in the literature regarding the relationship between CMV viral suppression and SNHL. In this study, we investigated hearing features in a pediatric cohort with cCMV infection, and explored the prognostic determinants of the hearing outcomes.

## Material and methods

### Patient recruitment and classification

Infants who were diagnosed with cCMV infection at a tertiary hospital (National Taiwan University Hospital) or were referred to this hospital because of cCMV infection between 2014 and 2019 were enrolled prospectively. Congenital CMV infection was identified by detection of the virus in urine, saliva, or blood during the first 3 weeks of life^15,24^. These infants were tested for cCMV infection through a recently implemented newborn cCMV screening program^25^ or they were tested because of clinical symptoms that indicated cCMV infection. All infants who were referred from other hospitals were subjected to viral examinations to reconfirm the diagnosis. Written informed consent was obtained from the parents of all infants. The study was approved by the Research Ethics Committees of the Taipei City Hospital and the National Taiwan University Hospital [201605089RINC], and the Research Ethics Committees of National Health Research Institute [201803092RINB]. All methods were performed in accordance with the relevant guidelines and regulations.

At enrollment, all children received audiologic assessments, blood cell and platelet counts, blood biochemistry, brain transfontanellar ultrasonography, abdominal ultrasonography, neurologic examinations, visual assessments, and blood CMV viral load determination during the newborn period. Brain MRI was performed based on clinicians’ decision. The children were categorized into three groups according to their symptoms at birth. Neonates were classified as symptomatic when one or more of the following symptoms were found after neonatal examinations: petechiae, jaundice with conjugated hyperbilirubinemia (total bilirubin > 1 mg/dL), hepatosplenomegaly, thrombocytopenia (platelet count < 144 k/μL), chorioretinitis, seizures, microcephaly, and intracranial calcifications. A neonate without apparent abnormalities suggestive of cCMV disease, but having SNHL was classified as asymptomatic with isolated hearing loss. The other neonates without apparent abnormalities or SNHL were classified as asymptomatic with normal hearing^26^.

### Newborn hearing screening

All subjects in this study received a two-step hearing screening at birth using automated auditory brainstem response (AABR). Those failing to pass both steps of the screening, in either or both ears, or those who tested positive for CMV at birth were scheduled for an additional outpatient hearing screening test using AABR at 1 month of age.

### Virologic tests for CMV

Congenital CMV infection was identified by detection of the virus in urine, saliva, or blood during the first 3 weeks of life. CMV detection was performed with a quantitative real-time PCR assay with FRET hybridization probes to detect glycoprotein B of CMV. The lower limit of detection, estimated with a CMV construct, was 10 cp/ml^25^.

The blood CMV viral load was evaluated every 6–12 months, or at a shorter interval whenever clinically indicated, to monitor viral suppression. The first viral load detected in the peripheral blood during the first month of life, before the administration of antiviral medication, was coined as “the initial viral load”.

### Audiologic assessments

All patients underwent a comprehensive audiologic assessment at 3 months^14,25,27^. The comprehensive audiologic assessment included behavioral testing in a sound field, testing of distortion product otoacoustic emissions, and testing of auditory brainstem response under sedation, to evaluate the hearing thresholds at 0.5, 1, 2, and 4 kHz in both ears. The average hearing threshold was calculated at these four frequencies (0.5, 1, 2, and 4 kHz), and was categorized as mild (26–40 dB hearing level, dB HL), moderate (41–70 dB HL), severe (71–95 dB HL), or profound (> 95 dB HL) hearing loss. For patients with bilateral SNHL, the hearing threshold was represented by audiometric data of the better ear^28^.

The audiologic assessments were then conducted every 6–12 months, and could be intensified depending on the clinical condition of the children^25^. Non-sedative behavioral hearing tests, whenever feasible, were employed to replace sedative hearing tests, as the cooperativity of the children increased with age. Otoscopic examination and tympanometry were also performed regularly to exclude middle ear disorders. Progressive SNHL was defined as worsening of auditory thresholds of ≥ 10 dBHL. Late-onset SNHL was diagnosed when a patient with normal hearing at birth developed hearing loss during follow-up^29^.

### Other clinical assessments

In addition to the audiologic assessments, infants with cCMV infection also underwent other clinical evaluations, including blood cell counts, blood biochemistry, neurologic assessments, and visual assessments, during every medical check-up as pertinent to their clinical symptoms. All participants were under surveillance for more than 1 year.

### Statistical analyses

The hearing outcomes in the children with cCMV infection were compared according to the presence of symptoms at birth, NHS results, laterality of the affected ears, and viral profiles. Fisher’s exact test was utilized to make between-group comparisons of categorical data, and Wilcoxon's rank-sum test for continuous data. All analyses were conducted using STATA software, version 11.0 (Stata Corp LLC, College Station, TX). All tests were 2-tailed and differences were reported as significant if the P-value was less than 0.05.

## Results

### Demographic data

Altogether, 39 patients were included in this study, including 25 males (64.1%) and 14 females (35.9%). Sixteen patients were recruited through our recently implemented newborn cCMV screening program ^25^, and the other 23 were enrolled through the clinical sessions that followed the diagnoses of cCMV infections by the hospital pediatricians. The median and mean ages of the patients at enrollment were 1 and 2.8 months, respectively. The median and mean follow-up periods were 4 and 3.7 years, respectively, including 2–3 years in 6 patients (15.4%), 3–4 years in 10 patients (25.6%), 4–5 years in 17 patients (43.6%), 5–6 years in 3 patients (7.7%), and > 6 years in 3 patients (7.7%). Eleven patients had received brain MRI. An overview of their baseline demographic and clinical characteristics is presented in Table 1.

**Table 1:** Demographic characteristics of the 39 patients with congenital cytomegalovirus infection.

	No. of patients	(%)
Total	39	100%
Male	25	64.1%
Asymptomatic with normal hearing	20	51.3%
Asymptomatic with isolated hearing loss	9	23.1%
Symptomatic at birth	10	25.6%
**Newborn hearing screen**		
Passed	26	66.7%
Failed	13	33.3%
**Hearing**		
Normal	23	59.0%
SNHL	16	41.0%
Unilateral SNHL	6	15.4%
Bilateral SNHL	10	25.6%

SNHL: Sensorineural hearing loss.

Among the 39 patients, 26 (66.7%) passed NHS at birth, 13 (33.3%) failed NHS. All the 13 patients who failed NHS were confirmed to have SNHL later on diagnostic audiologic examinations. Ten patients (25.6% ) were classified as symptomatic at birth, 9 patients (23.1%) were asymptomatic with isolated hearing loss, and the remaining 20 patients (51.3%) were asymptomatic with normal hearing. A total of 16 patients (41.0%) were confirmed to have SNHL, including 6 with unilateral (15.4%) and 10 with bilateral (25.6%) SNHL. Among these 16 patients, 3 passed NHS at birth, but developed SNHL during the follow-up period, suggesting late-onset SNHL. These 3 patients were confirmed to have hearing loss at 3, 8, and 15 months old, respectively.

Of the 10 symptomatic patients, 4 presented with SNHL as well as other symptoms at birth, and 6 presented with symptoms other than SNHL at birth. Among these 6 patients with symptoms other than SNHL at birth, 2 patients developed late-onset SNHL during the follow-up period. In addition, of the 10 symptomatic patients, three patients displayed abnormal brain magnetic resonance imaging findings.

### Presence of cCMV symptoms at birth vs. hearing outcomes

The hearing outcomes of each group of patients at their last visit to our clinic are shown in Table 2. Of the 20 asymptomatic patients with normal hearing, 9 asymptomatic patients with isolated hearing loss, and 10 symptomatic patients, 1 (5%), 9 (100%), and 6 (60%) patients had SNHL, respectively, showing a significant difference among the three groups (Fisher’s exact test, *P* < 0.001).

**Table 2:** Comparison of hearing outcomes according to the presence of symptoms at birth and NHS results.

	Asymptomatic with normal hearing	Asymptomatic with isolated HL	Symptomatic	P value	Pass NHS	Fail NHS	**P value**
Patients	20	9	10		26	13	
Normal hearing	19 (95%)	0	4 (40%)	P < 0.001^a^	23 (88.5%)	0	P < 0.001^a^
SNHL	1 (5%)	9 (100%)	6 (60%)		3 (11.5%)	13(100%)	
Severity of SNHL (ears)	2	15	9		6	20	
Mild HL	2 (100%)	2 (13.3%)	0	P = 0.221^a^	2 (33.3%)	2 (10.0%)	P = 0.305^a^
Moderate HL	0	3 (20%)	2 (22.2%)		0	5 (25.0%)	
Severe HL	0	4 (26.7%)	2 (22.2%)		0	2 (10.0%)	
Profound HL	0	6 (40%)	5 (55.6%)		4 (66.7%)	11 (55.0%)	

SNHL: Sensorineural hearing loss; HL: Hearing loss; NHS: Newborn hearing screen.

^a^: Fisher’s exact test.

Of the 16 patients who developed SNHL, 3 asymptomatic patients with isolated hearing loss and 3 symptomatic patients were affected unilaterally, whereas the other 10 patients were affected bilaterally. Of the 26 affected ears, the distribution of SNHL severity did not differ among the three groups (Table 2; Fisher’s exact test, P = 0.221).

### NHS results vs. hearing outcomes

The hearing outcomes of patients who passed NHS and of those who failed NHS are compared in Table 2. Among the 26 patients who passed NHS at birth, 3 (11.5%) developed SNHL and all were bilaterally affected; whereas among the 13 patients who failed NHS at birth, all (100%) had SNHL, including 6 with unilateral and 7 with bilateral SNHL. Ear-wise distribution of severity is summarized in the Table 2.

Of the 10 patients with bilateral SNHL, 2, 3, 1, and 4 patients had mild, moderate, severe, and profound SNHL, respectively. Seven patients used hearing aids, whereas the other three patients with bilateral profound SNHL underwent cochlear implantation because of limited benefits with hearing aids. Of these three patients, one patient exhibited good auditory speech performance after cochlear implantation, whereas the other two showed less favorable outcomes because of the involvement of the central nervous system by cCMV infection: both patients had seizure after birth, and one of them presented with lissencephaly with polymicrogyria on brain MRI .

### Laterality vs. hearing outcomes

The severity of SNHL in the unilaterally affected ears (n = 6, from 6 unilateral patients) and in the bilaterally affected ears (n = 20, from 10 bilateral patients) is shown in Table 3. Of the 6 unilaterally affected ears, 2 (33.3%) and 4 (66.7%) had severe and profound SNHL, respectively; whereas, out of the 20 bilaterally affected ears, 4 (20%), 5 (25%), 4 (20%), and 7 (35%) had mild, moderate, severe, and profound SNHL, respectively. There was no statistically significant difference in the distribution of SNHL severity between the two groups (Fisher’s exact test, P = 0.353).

**Table 3:** Comparison of SNHL severity according to the laterality of the affected ears.

	No. of affected ears	Severity of SNHL				*P* value
		Mild	Moderate	Severe	Profound	
Unilateral	6	0	0	2 (33.3%)	4 (66.7%)	*P* = 0.353^a^
Bilateral	20	4 (20%)	5 (25%)	4 (20%)	7 (35%)	

SNHL: Sensorineural hearing loss.

^a^ : Fisher’s exact test.

### Initial blood viral loads vs. symptoms at birth, NHS results, and development of SNHL

We then compared the initial viral loads according to the presence of symptoms at birth, NHS results, and the development of SNHL (Table 4). The viral loads during the first month of life could be ascertained in 21 out of the 39 subjects. Due to the limited sample size, we combined asymptomatic patients with normal hearing and asymptomatic patients with isolated hearing loss into a single “asymptomatic” group. The viral loads were lower in children with asymptomatic cCMV infection at birth than in those with symptomatic infection (median = 0 vs. 1100 cp/mL, mean = 695 vs. 2396 cp/mL, Wilcoxon's rank-sum test, P = 0.040); in children who passed NHS than in those who failed NHS (median = 0 vs. 1126 cp/ml, mean = 715 vs. 2738 cp/mL, Wilcoxon's rank-sum test, P = 0.053); and in children who did not develop SNHL than in those who developed SNHL (median = 0 vs. 1126 cp/ml, mean = 715 vs. 2738 cp/mL, Wilcoxon's rank-sum test, P = 0.053).

**Table 4:** Comparison of initial viral loads in congenital cytomegalovirus infection patients with different presentations and outcomes.

Patients	Initial viral load, cp/mL			No. of early suppression (%)	P value
	Median (IQR)	Mean (SE)	*P* value		
Asymptomatic (n = 16)	0 (710)	695 (336)	P = 0.040^a^	11 (68.8%)	P = 0.012^b^
Symptomatic (n = 5)	1100 (4229)	2396 (1533)		0 (0%)	
Pass NHS (n = 17)	0 (1100)	715 (317)	P = 0.053^a^	11 (64.7%)	P = 0.035^b^
Fail NHS (n = 4)	1126 (4194)	2738 (1926)		0 (0%)	
Normal hearing (n = 17)	0 (1100)	715 (317)	P = 0.053^a^	11 (64.7%)	P = 0.035^b^
SNHL (n = 4)	1126 (4194)	2738 (1926)		0 (0%)	

IQR: Interquartile range; NHS: Newborn hearing screen; SNHL: Sensorineural hearing loss.

^a^: Wilcoxon's rank-sum test; ^b^: Fisher’s exact test; SE: stanard error.

Consistent with previous reports^19^, the blood viral load became undetectable without antiviral therapy in certain subjects during the first month of life. The percentages of this “spontaneous early viral suppression” were significantly higher in children with asymptomatic infection at birth than in those with symptomatic infection (68.8% vs. 0%), in children who passed NHS than in those who failed NHS (64.7% vs. 0%), and in children who did not develop SNHL than in those who developed SNHL (64.7% vs. 0%) (Table 4; Fisher’s exact test, all *P* < 0.05).

Ten (25.6%) of the 39 subjects received antiviral therapy to control disease progression, including 4 with intravenous ganciclovir, 4 with oral valganciclovir, and 2 with a combination of both. Of the 10 subjects, 9 received antiviral treatment for symptomatic disease (i.e., cCMV-related symptoms in addtion SNHL), whereas the other one for progression of SNHL. The duration of antiviral treatment ranged from 3 weeks to 6 months, as per the decision of the pediatricians, with a mean duration of 4 months. The initial viral loads were higher in patients who received antiviral therapy than in those who did not (median = 1100 vs. 0 cp/ml, Wilcoxon's rank-sum test, P = 0.0497). However, this finding needs to be interpreted with caution because of the limited sample size. The blood viral loads decreased significantly in all the 10 patients after antiviral therapy, with the mean viral load decreasing from 5227 cp/mL before treatment to 38 cp/mL after treatment.

### Special cases

**Figure 1:**
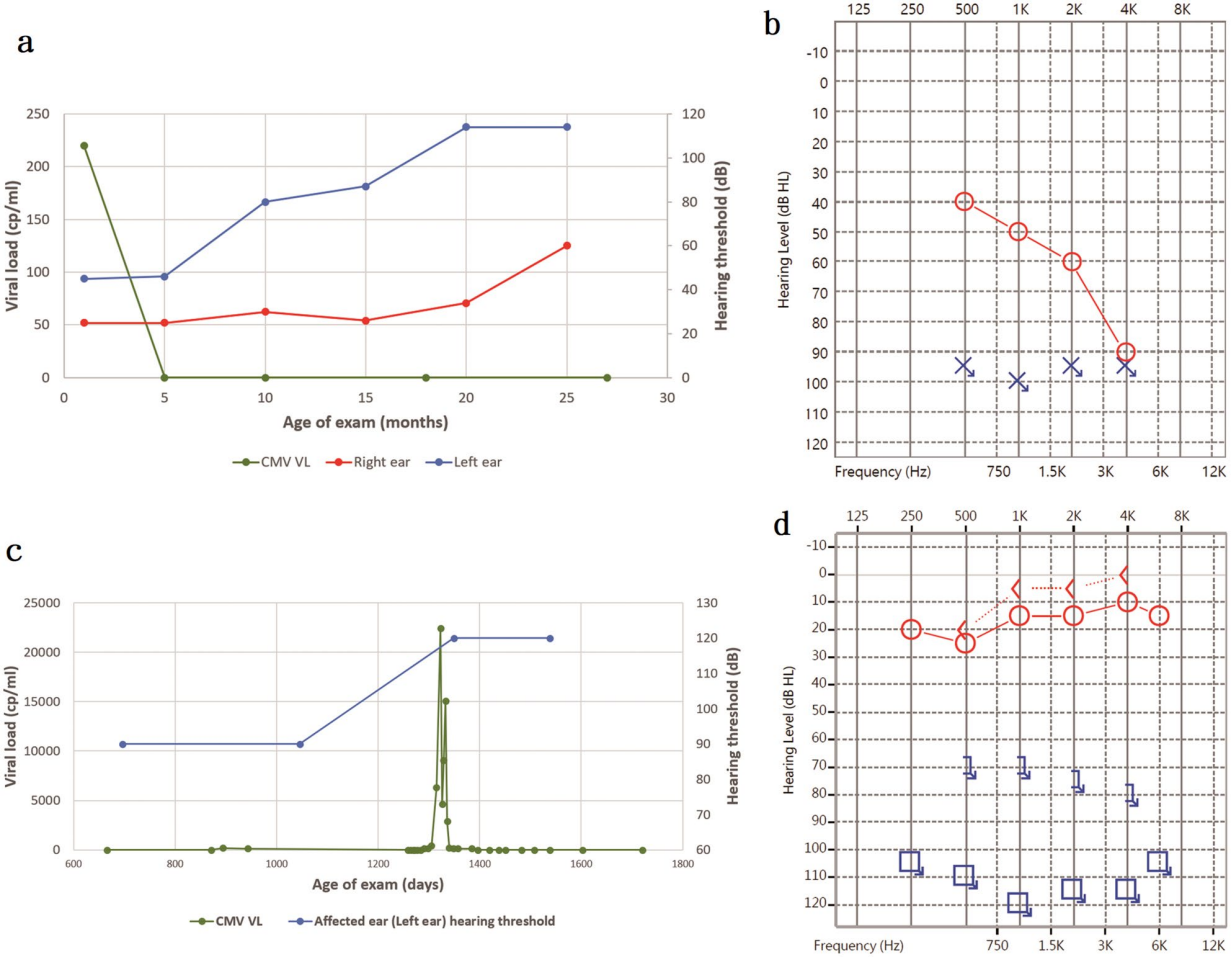
The relation between viral load and hearing loss in two special cases. (**a**) Case A, who was symptomatic at birth, developed progressive bilateral sensorineural hearing loss (SNHL) even after successful serum viral load suppression at 5 m. (**b**) The latest audiogram of Case A at 2 y 1 m. Audiogram showing profound hearing loss of left ear and moderate hearing loss of right ear. Air conduction: “O” for right ear, “X” for left. “↘”: No response to the loudest sounds. (**c**) Case B was a patient of symptomatic congenital CMV infection with left SNHL at birth. She was diagnosed as having activated PI3K-delta syndrome at 2 y, and received peripheral blood stem cell transplantation. The left ear SNHL deteriorated concurrently with the flare-up of viral activity. (**d**) The latest audiogram of Case B at 4 y 2 m, showing left profound hearing loss and normal hearing in the right ear. Air conduction: “O” for right ear, “□” for left. Bone conduction: “ < ” for right ear, “]” for left. “↘”: No response to the loudest sounds.

To further investigate the correlation between viral activity and SNHL, herein we highlighted two special cases in our cohort. Case A had an asymptomatic cCMV infection with isolated SNHL at birth. Serum viral suppression was noted at 5 months of age (Fig. 1a), and CMV viral activity remained undetectable during serial follow-ups. However, serial audiologic examinations showed progressive bilateral SNHL (Fig. 1a,b). SNHL of left ear, which was congenital and moderate (~ 45 dB HL) at birth, progressed to a profound degree (> 110 dB HL). In the meantime, the right ear, which was normal at birth developed delayed-onset moderate SNHL (up to ~ 60 dB HL).

Case B had an asymptomatic cCMV infection with isolated unilateral left SNHL at birth. She was diagnosed as having activated PI3K-delta syndrome, an immunodeficiency disease, at two years of age. This child received a peripheral blood stem cell transplantation between 1270 and 1400 days of age (41 and 45 months). The blood CMV viral load increased during the transplantation period, and the left ear SNHL deteriorated concurrently (Fig. 1c). The blood CMV viral load became undetectable after transplantation, and the hearing threshold of the left ear also stabilized (Fig. 1c,d). During this period, no ototoxic medications were administered. Progressive SNHL in this patient may have been related to the flare-up of the viral activity.

## Discussion

In this study, we demonstrated that the presence of symptoms at birth was associated with the hearing outcome of cCMV infection, whereas the uni- or bi-laterality of the affected ears was not. NHS was a reliable tool for early detection of SNHL when infants fail the test, while a pass of NHS was not a guarantee for not having SNHL. Our analyses of the initial viral loads, and the time-sequence between viral load flare-up and SNHL deterioration, suggested that CMV viral activity might also be associated with the development of SNHL.

In our cohort, 5% (1/20) of asymptomatic newborns and 100% (9/9) of asymptomatic newborns with isolated hearing impairment developed SNHL (Table 2). Together, 34.5% (10/29) of grossly asymptomatic cCMV infection neonates developed SNHL. The proportion was higher than that reported previously (6%–23%)^8,10,11,13,14,30,31^. A significant number of our cCMV cases were identified among newborns with abnormal NHS results. In earlier studies, SNHL could usually not be detected at birth before the advent of universal NHS. Therefore, children with cCMV infection and isolated hearing impairment might not be detected and grouped as such in some earlier studies. In addition, the higher percentages of SNHL observed in our study might also be attributed to the different thresholds used to define SNHL (25 dB HL in our study, and 25 dB HL^13,30^ or 30 dB HL^11^ in previous studies). Besides, the small sample size and selection bias may have also contributed to this higher proportion of SNHL in asymptomatic infants in this study.

Notably, all 13 cCMV-infected infants who failed NHS were later confirmed as having SNHL. This finding was consistent with a previous study documenting that children with cCMV infection who failed NHS were more likely to develop SNHL^10^. Several prior studies emphasized the compensatory role of newborn cCMV screening for NHS by identifying children who passed NHS but were at risk for SNHL^9,27,32,33^. Intensive follow-up is indicated in all children with cCMV, no matter they failed NHS or not ^9,11,27,34^.

Previous studies reported that asymptomatic newborns tended to develop unilateral SNHL (57%-71.4%)^11,15^, whereas symptomatic newborns could develop either unilateral or bilateral SNHL^10,11,15^. Of the six symptomatic patients with SNHL in our cohort, three (50%) had bilateral SNHL, and three (50%) had unilateral SNHL. Of the nine asymptomatic patients with isolated SNHL, six (66.7%) had bilateral SNHL, and three (33.3%) had unilateral SNHL. We did not observe an association between the presence of cCMV symptoms and the uni- or bi-laterality of SNHL in this study.

Interestingly, there was no difference between SNHL severity in the unilaterally-affected ears (n = 6, from 6 unilateral patients) and that of the bilaterally-affected ears (n = 20, from 10 bilateral patients) (Table 3). In other words, although the extent of disease appears more limited in unilateral infection than that in bilateral infection, once the ear is infected, the pathology in the unilaterally-affected ears might be as severe as that in the bilaterally-affected ears. It has been reported that in animal models, CMV may spread into the inner ear through modiolar blood vessels, spiral ganglions and perilymph route^35,36^, and cause direct cytopathic or localized inflammatory respose^35–41^. These pathogenetic mechanisms indicate that the involvement of inner ear structure by cCMV infection in each ear might be an independent event, which is consistent with our observation.

It has been documented that symptomatic newborns have higher blood viral loads than asymptomatic newborns^18,20,21,42^. Previous studies also showed that an elevated blood viral load during early infancy might increase the risk for SNHL in infants with asymptomatic cCMV infection^18–20^, but the results regarding the association between viral load and SNHL in symptomatic infants were variable^18,20–23^. Parallel to these clinical observations, it was demonstrated in an animal study that the spread of cCMV infection into the inner ear of guinea pigs was preceded by viremia^35^. In this study, our results revealed that the initial viral load in symptomatic newborns was significantly higher than that in asymptomatic newborns. Patients who failed NHS and patients who proved to have SNHL also tend to have higher initial viral loads after birth. Moreover, spontaneous viral suppression during the first month of life was seen exclusively in patients who were asymptomatic at birth, having a higher rate of passing NHS, and a lower rate of developing SNHL. However, these findings should be interpreted carefully due to limited case number. Previous results regarding the association between viral load and SNHL were variable^20,21^. The viral suppression in blood may not represent viral clearance at the other body sites such as inner ear or CNS, where the antiviral concentration is less than plasma levels. More studies in larger study populations are necessary before making any conclusions on association between viral load and hearing outcome.

The two special cases we described in this study also provided important insight into the interaction between viral activity and the development of SNHL. As demonstrated in Case A, it is notable that SNHL could deteriorate even after suppression of serum CMV was achieved. On the other hand, we observed in Case B that SNHL might also deteriorate with the flare-up of serum CMV load. Based on these findings, it is recommended that children with cCMV infection should receive regular audiologic examination even after serum viral suppression. Moreover, if viral activity flares up due to certain immunological factors, the physicians should also be aware of the risk of progression of SNHL.

By analyzing the interplay between longitudinal viral profiles and multiple hearing features in a cohort recruited from a single institute, this study identified several prognostic indicators for the hearing outcomes of cCMV infection. However, some limitations of this study merit discussion. First, the number of patients in our cohort was limited. A more extensive series will possibly identify the predictive factors for cCMV-related SNHL with greater precision. Second, observations in this study were of an “associative” nature and not of a “causative” nature. Further research is required to elucidate the pathogenetic mechanisms of SNHL induced by cCMV infection. Third, as we enrolled subjects from a tertiary care hospital, selection bias may have arisen, which was evidenced by a higher percentage of symptomatic infants in our cohort.

## Conclusion

The presence of cCMV-related symptoms (e.g., petechiae, jaundice, hepatosplenomegaly, thrombocytopenia, chorioretinitis, seizures, and microcephaly) at birth, the failure on NHS, and viral activity are the main prognostic factors that predict the development of SNHL in infants with cCMV infection, whereas being bi- or unilaterally affected does not. Despite the correlation between viral activity and the hearing outcomes, regular audiologic examinations are warranted even after CMV DNAemia clearance, especially for children with serum CMV load flare-up due to certain immunological causes. However, cautions should be taken when interpreting the results as our study scale is small. Researches with larger study populations is necessary to further refine the prognostics of cCMV-related SNHL.
